# Four-dimensional NOE-NOE spectroscopy of SARS-CoV-2 Main Protease to facilitate resonance assignment and structural analysis

**DOI:** 10.5194/mr-2-129-2021

**Published:** 2021-04-13

**Authors:** Angus J. Robertson, Jinfa Ying, Ad Bax

**Affiliations:** Laboratory of Chemical Physics, National Institute of Diabetes and Digestive and Kidney Diseases, National Institutes of Health, Bethesda, MD 20892, USA

## Abstract

Resonance assignment and structural studies of larger proteins by nuclear magnetic resonance (NMR) can be challenging when exchange broadening, multiple stable conformations, and ${}^{1}$ H back-exchange of the fully deuterated chain pose problems. These difficulties arise for the SARS-CoV-2 Main Protease, a homodimer of 2 × 306 residues. We demonstrate that the combination of four-dimensional (4D) TROSY-NOESY-TROSY spectroscopy and 4D NOESY-NOESY-TROSY spectroscopy provides an effective tool for delineating the ${}^{1}$ H– ${}^{1}$ H dipolar relaxation network. In combination with detailed structural information obtained from prior X-ray crystallography work, such data are particularly useful for extending and validating resonance assignments as well as for probing structural features.

## 1 Introduction

The extension of conventional two-dimensional ${}^{1}$ H– ${}^{1}$ H NMR spectroscopy of natural proteins (Wüthrich, 1986) to three-dimensional (3D) homonuclear NMR experiments offered the ability to simplify spectral analysis by removing resonance overlap (Vuister et al., 1988; Oschkinat et al., 1988) and by providing access to a direct, more detailed analysis of ${}^{1}$ H– ${}^{1}$ H dipolar cross-relaxation networks. In particular, the homonuclear 3D NOE-NOE experiment (Boelens et al., 1989; Breg et al., 1990) not only decreased resonance overlap, it also directly elucidated spin-diffusion pathways. This information complemented and validated the elegant relaxation matrix analysis of spin diffusion (Boelens et al., 1988).

Such homonuclear ${}^{1}$ H 3D experiments and analysis strategies were soon followed by a myriad of heteronuclear 3D experiments that required isotopic enrichment and therefore cloning and bacterial overexpression (Marion et al., 1989b; Zuiderweg and Fesik, 1989; Ikura et al., 1990; Marion et al., 1989a; Wagner, 1993). Most of these heteronuclear experiments simply served to disperse the regular ${}^{1}$ H– ${}^{1}$ H 2D spectrum into a third dimension, thereby removing spectral overlap but providing little or no new information on the all-important ${}^{1}$ H– ${}^{1}$ H spin-diffusion pathways. The 3D NOESY-HMQC experiment (Marion et al., 1989b; Zuiderweg and Fesik, 1989) subsequently was extended to four dimensions (4D), thereby dispersing the conventional 2D ${}^{1}$ H– ${}^{1}$ H NOESY experiment into two additional dimensions that correspond to the chemical shifts of the nuclei to which each of the protons is covalently bound (Kay et al., 1990; Clore et al., 1991; Zuiderweg et al., 1991).

These multi-dimensional experiments provided a tremendous degree of spectral simplification, in particular after appropriate analysis software became available. However, it also quickly became clear that extension to large, slowly tumbling proteins was hampered by low signal to noise, caused by the relative inefficiency of the magnetization transfer steps when the dimensionality of a spectrum is increased. This decrease in sensitivity was remedied by generating the protein in a highly perdeuterated state while keeping the solvent-exchangeable backbone amide protons protonated (Torchia et al., 1988; Lemaster and Richards, 1988). Combining the perdeuteration approach with both the triple-resonance assignment strategy (Grzesiek et al., 1993) and the subsequently introduced powerful TROSY line-narrowing method (Pervushin et al., 1997) made it possible to assign and analyze the structure of quite large proteins, as exemplified by the 723-residue protein malate synthase G (Tugarinov et al., 2002, 2005a). The sensitivity gained by perdeuteration, enabling the recording of 4D ${}^{15}$ N-separated NOE spectra, also was key in solving the structure of a HIV-1 accessory protein that had been too challenging for analysis by more conventional methods (Grzesiek et al., 1995). Zhu and co-workers introduced a TROSY-NOESY-TROSY version of Grzesiek's 4D NOESY experiment which, illustrated for a partially deuterated 27 kDa protein, yielded further improved sensitivity and intrinsic ${}^{1}$ H and ${}^{15}$ N line widths (Xia et al., 2000). Their implementation relied on eight-step phase cycling, thereby limiting digitization of the time-domain data and unable to exploit the improved TROSY relaxation in the indirect dimensions. Diercks et al. (2010) introduced an elegant method to suppress diagonal signals from a 4D TROSY-NOESY-TROSY spectrum (Diercks et al., 2010), a particularly useful feature when spectral resolution is limited. However, we opted not to use this implementation as, at the long mixing times used, the diagonal resonances serve as convenient reference anchors during analysis and are sufficiently attenuated such as not to obscure nearby peaks in our 4D spectra that are of very high digital resolution.

In the present report, we merge the above-mentioned prior advances, 3D NOE-NOE and 3D ${}^{15}$ N-separated NOESY, into a 4D experiment. In combination with extensive perdeuteration and gradient-enhanced encoding to enable a four-step phase cycle as well as non-uniform sampling (NUS) (Rovnyak et al., 2004), the experiments take better advantage of the improved resolution afforded by 4D NMR. We demonstrate the utility of the experiments by applying them to the study of the main protease of SARS-CoV-2 (M ${}^{\mathrm{pro}}$ ), which is the virus responsible for coronavirus-2019 disease (COVID-19). M ${}^{\mathrm{pro}}$ , also known as 3CL ${}^{\mathrm{pro}}$ or Nsp5, is a homodimeric cysteine protease of 2 × 306 residues that does not have closely related mammalian homologues and is therefore an intense target for drug development, with a promising inhibitor now entered in a phase I clinical trial (Boras et al., 2021). Its NMR analysis is challenging, not only for its large size (67.6 kD), but also because of the presence of a minor conformer associated with the cis-isomer of one of its 13 Pro residues (P184), the difficulty in back-exchanging all backbone amide protons when the protein is expressed in ${}^{2}$ H ${}_{2}$ O, and the presence of intermediate timescale motions that lead to exchange broadening in the vicinity of the protein's active site. Here we focus on an enzyme variant where the catalytic Cys145 residue has been mutated to Ala (M ${}_{C145A}^{\mathrm{pro}}$ ), a construct that is stable for multiple weeks at the high concentrations required for NMR spectroscopy. The assignment process and a full structural analysis of the protein will be presented elsewhere. The focus of the present work is on technical innovations, including recording two types of 4D NOE-based NMR spectra that proved invaluable both for the validation of resonance assignments as well as the subsequent structural analysis.

## 2 Methods and experiments

### 2.1 Protein production

The gene encoding a C145A variant of M ${}^{\mathrm{pro}}$ (M ${}_{C145A}^{\mathrm{pro}}$ ) with an N-terminal affinity (and solubility) tag was synthesized by GenScript (USA) and then cloned into a Pet24a+ plasmid between BamH1 and Xho1 restriction sites. The fusion protein encoded for 6His tag – GB1–SG rich linker–TEV cleavage site – M ${}_{C145A}^{\mathrm{pro}}$ and was purified according to methods collectively developed by the COVID-19 NMR consortium (Altincekic, 2021). In brief, following cell culture and harvesting (see Supplement), the cell lysate was passed down a 6His-affinity column (IMAC) and eluted in a small volume; the solubility tag was cleaved off to generate a native N-terminus; the reaction mix was then passed through an IMAC column to remove uncleaved protein before size separation on a Sephadex G75 column. The resulting, extensively perdeuterated, ${}^{2}$ H, ${}^{15}$ N-M ${}_{C145A}^{\mathrm{pro}}$ homodimer was used for experiments and, throughout the recombinant protein expression, extensive care was taken to achieve a high ( ∼ 98 %) level of deuteration of the non-exchangeable hydrogens. For more details, see the Supplement.

### 2.2 Recording of NMR data

Spectra were acquired on a sample containing 1.8 mM (0.9 mM dimer) ${}^{2}$ H, ${}^{15}$ N-M ${}_{C145A}^{\mathrm{pro}}$ in 10 mM sodium phosphate, pH 7.0, 0.5 mM TCEP, 3 % v/v ${}^{2}$ H ${}_{2}$ O and 0.3 mM sodium trimethylsilylpropanesulfonate (DSS; as an internal chemical shift reference), in a 300 µ L Shigemi microcell. All experiments were recorded at 25 ${}^{\circ }$ C on an 800 MHz Bruker Avance III spectrometer, equipped with a 5 mm TCI probe containing a triple-axis gradient accessory, and running TopSpin software version 3.1.

**Figure 1:**
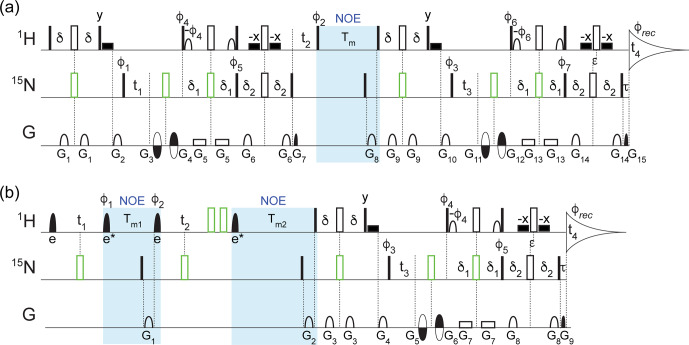
Pulse schemes for four-dimensional **(a)** TROSY-NOESY-TROSY and **(b)** NOESY-NOESY-TROSY experiments. The filled and open rectangular bars on the ${}^{1}$ H and ${}^{15}$ N channels represent 90 and 180 ${}^{\circ }$ pulses, respectively. The open bars in green represent composite 180 ${}^{\circ }$ pulses consisting of 90 ${}^{\circ }$ ${}_{x}$ 207 ${}^{\circ }$ ${}_{y}$ 90 ${}^{\circ }$ ${}_{x}$ for ${}^{1}$ H and 90 ${}^{\circ }$ ${}_{x}$ 216 ${}^{\circ }$ ${}_{y}$ 90 ${}^{\circ }$ ${}_{x}$ for ${}^{15}$ N. The filled shaped ${}^{1}$ H pulses correspond to selective EBURP2 (labeled e) and time-reversed EBURP2 (labeled e*) pulses (Geen and Freeman, 1991), while the open ${}^{1}$ H-shaped pulses represent 90 ${}^{\circ }$ water-flipback pulses (center lobe of a sinc profile, 1.1 ms duration at 800 MHz) (Grzesiek and Bax, 1993). The wide filled rectangular boxes denote 90 ${}^{\circ }$ water-flipback pulses (also 1.1 ms duration at 800 MHz). Unless indicated otherwise, all pulses were applied along x. The following delays were used for the initial INEPT and TROSY transfers: δ = 2.1 ms, $\delta_{1}$ = 2.1 ms, $\delta_{2}$ = 2.5 ms. The ${}^{1}$ H chemical shift evolution during the delay τ = 0.181 ms was compensated by offsetting the last pair of ${}^{1}$ H and ${}^{15}$ N 180 ${}^{\circ }$ pulses by ε=τ/2 to avoid the linear phase error in the $t_{4}$ dimension. **(a)** For the TROSY-NOESY-TROSY: NOE mixing time $T_{m}$ = 200 ms; phase cycling: $\varphi_{1}$ = y, $\varphi_{2}$ = x , x , -x , -x , $\varphi_{3}$ = y , -y , $\varphi_{4}$ = y , $\varphi_{5}$ = y , $\varphi_{6}$ = y , $\varphi_{7}$ = y , $\varphi_{\mathrm{rec}}$ = y , -y , -y , y ; gradients were sine-bell or rectangular shaped (as depicted in the figure) with durations G ${}_{1,2,3,4,5,6,7,8,9,10,11,12,13,14,15}$ = 0.977, 1.2, 0.4, 0.4, 0.986, 0.977, 0.081, 1.7, 0.977, 1.2, 0.4, 0.4, 0.986, 0.977, and 0.081 ms, z strengths of 21.7, 28.7, - 25.9, 32.9, 2.1, 25.9, 29.4, 30.8, 21.7, 28.7, - 31.5, 38.5, 2.1, 25.9, and 35.0 G cm ${}^{-1}$ , and additional x and y strengths of - 22.5, 27.5, and 25.0 G cm ${}^{-1}$ for G ${}_{11}$ , G ${}_{12}$ , and G ${}_{15}$ , respectively. The duration of decoding pulses G ${}_{7}$ (G ${}_{15}$ ) was empirically optimized for maximum signal and can differ from the theoretical value derived from the gyromagnetic ratios of ${}^{15}$ N and ${}^{1}$ H and the encoding pulses G ${}_{3}+G_{4}$ (G ${}_{11}+G_{12}$ ) by several microseconds due to rise and fall times of short gradient pulses. Quadrature detection in $t_{3}$ ( $t_{1}$ ) was achieved using the Echo-AntiEcho scheme (Kay et al., 1992) by inverting the encoding gradient G ${}_{11}$ and G ${}_{12}$ (G ${}_{3}$ and G ${}_{4}$ ) together with $\varphi_{6}$ and $\varphi_{7}$ ( $\varphi_{4}$ and $\varphi_{5}$ ) to obtain the second FID for every $t_{3}$ ( $t_{1}$ ) increment. The $t_{2}$ dimension was acquired using States-TPPI by incrementing $\varphi_{2}$ by 90 ${}^{\circ }$ . **(b)** For the NOESY-NOESY-TROSY, the selective EBURP2 and time-reversed EBURP2 pulses have a duration of 1.0 ms at 800 MHz, centered at 8.3 ppm, thereby exciting the amide protons downfield from the water resonance without perturbing any upfield exchangeable protons, or residual aliphatic protons resulting from imperfect perdeuteration. NOE mixing times, $T_{m1}$ = 50 ms; $T_{m2}$ = 300 ms. Phase cycling: $\varphi_{1}$ = x , x , -x , -x , $\varphi_{2}$ = x-π/4 , $\varphi_{3}$ = y , -y , $\varphi_{4}$ = y , $\varphi_{5}$ = y , $\varphi_{\mathrm{rec}}$ = y , -y , -y , y . Gradients were sine-bell or rectangular shaped with durations G ${}_{1,2,3,4,5,6,7,8,9}$ = 1.7, 1.2, 0.977, 1.2, 0.4, 0.4, 0.986, 0.977, and 0.081 ms, z strengths of 20.3, 30.8, 21.7, 28.7, - 31.5, 38.5, 2.1, 25.9, and 35.0 G cm ${}^{-1}$ , and additional x and y strengths of - 22.5, 27.5 and 25.0 G cm ${}^{-1}$ for G ${}_{5}$ , G ${}_{6}$ , and G ${}_{9}$ , respectively. The duration of G ${}_{9}$ was empirically optimized for maximum signal. Quadrature detection in $t_{3}$ was achieved using the Echo-AntiEcho scheme by inverting the encoding gradient G ${}_{5}$ and G ${}_{6}$ together with the $\varphi_{4}$ and $\varphi_{5}$ to obtain the second FID for every $t_{3}$ increment, while States-TPPI was used to obtain quadrature in the $t_{1}$ dimension by incrementing the $\varphi_{1}$ pulse phase by 90 ${}^{\circ }$ and for $t_{2}$ by incrementing $\varphi_{2}$ by 90 ${}^{\circ }$ . Pulse sequence code and parameter files can be downloaded from https://zenodo.org/record/4625615.

Considering that the measurements are carried out for perdeuterated protein, the spectral windows in the indirect ${}^{1}$ H dimensions were limited to the region downfield from the water resonance, and EBURP2 pulses (Geen and Freeman, 1991) were used where needed to prevent excitation of protons outside of this region. For the 4D TROSY-NOESY-TROSY experiment (Fig. 1a), only a small fraction (0.54 %) of the full data matrix, consisting of 1536* ( ${}^{1}$ H, $t_{4}$ , 119.8 ms) × 90* ( ${}^{15}$ N, $t_{3}$ , 35.1 ms) × 91* ( ${}^{1}$ H, $t_{2}$ , 20.0 ms) × 90* ( ${}^{15}$ N, $t_{1}$ , 35.1 ms) complex points, was recorded (Table S1 in the Supplement) using nonuniform sampling for the indirect dimensions. Selection of the sampled data points was unweighted, randomly distributed, and without time ordering. The sampling schedule (included in the uploaded data sets; see Data availability) was automatically generated by Topspin 3.1 when the experiment started, with the random seed left to its default value (54 321) and the $T_{2}$ for each indirect dimension set to 1 s to avoid significant weighting of the sampling schedule. Specifically, a total of 31896 FIDs along the directly detected $t_{4}$ dimension were recorded. Using four scans per FID for phase cycling and an interscan delay of 2.07 s, the total measurement time was approximately 88 h, but 3-fold shorter would have sufficed (see Concluding remarks).

Nonstandard processing was needed for the TROSY-NOESY-TROSY experiment because the spectrum was recorded with sensitivity-enhanced gradient selection in the ${}^{15}$ N $t_{1}$ evolution period that preceded the NOE mixing (Xia et al., 2000). Specifically, the 4D NUS data set was first sorted and expanded according to the sampling schedule using the *nusExpand.tcl* script within the NMRPipe software package (Delaglio et al., 1995). The expanded data were then converted to the NMRPipe format, with the quadrature mode for $t_{3}$ set to *Echo-AntiEcho*, while the quadrature mode for $t_{1}$ was temporarily set to *Complex*. After the conversion, the 4D matrix needs to be transposed to enable use of the NMRPipe macro *bruk_ranceA.M* to correctly reshuffle the data, turning the phase-modulated $t_{1}$ dimension into conventional amplitude-modulated data prior to processing as regular, complex data. This transposition is accomplished by reading in the NMRPipe-formatted matrix with the z axis along the $t_{2}$ dimension, application of the macro, and restoring the data to its original axis order prior to regular processing, with the full script included with the raw time-domain data sets (see Data availability). For the processing, the direct dimension was apodized with a squared, shifted sine-bell window, spanning from 72 to 176.4 ${}^{\circ }$ , whereas an additional 15 Hz exponential line broadening was used to better match the apodization window to the natural decay of the signal, thereby improving the signal-to-noise ratio ( S/N ). This was followed by zero filling and Fourier transformation. Subsequently, the indirect data points that were not experimentally sampled were reconstructed using the SMILE (Ying et al., 2017) program, and the reconstructed data were further processed in NMRPipe. To enhance the spectral resolution, by default the acquisition times in all indirect dimensions were extended by 50 % during the SMILE reconstruction, leading to an effective sampling sparsity of 0.16 %. The data matrix for the final reconstructed 4D spectrum consists of 614 ( ${}^{1}$ H, $F_{4}$ , 6.3 Hz per point) × 512 ( ${}^{15}$ N, $F_{3}$ , 5.0 Hz per point) × 512 ( ${}^{1}$ H, $F_{2}$ , 8.9 Hz per point) × 512 ( ${}^{15}$ N, $F_{1}$ , 5.0 Hz per point) real points (see Table S1).

The full time-domain data matrix of the 4D NOESY-NOESY-TROSY experiment (Fig. 1b) consists of 1536* ( ${}^{1}$ H, $t_{4}$ , 95.8 ms) × 90* ( ${}^{15}$ N, $t_{3}$ , 35.1 ms) × 60* ( ${}^{1}$ H, $t_{2}$ , 12.0 ms) × 60* ( ${}^{1}$ H, $t_{1}$ , 12.0 ms) complex points (Table S1). An unweighted, random NUS sampling scheme with a sparsity of 1.69 % (corresponding to 43 856 $t_{4}$ FIDs) was used to record a small subset of the data points. Using an interscan delay of 1.77 s and four scans per FID, the total experimental time was approximately 110 h, but 3-fold shorter would have sufficed (see Concluding remarks). The data were processed and reconstructed in the same manner as described above with 50 % extension of all indirect dimensions during the SMILE reconstruction, resulting in an effective sparsity of 0.50 % and a final spectral matrix size of 492 ( ${}^{1}$ H, $F_{4}$ , 7.8 Hz per point) × 512 ( ${}^{15}$ N, $F_{3}$ , 4.7 Hz per point) × 512 ( ${}^{1}$ H, $F_{2}$ , 13.9 Hz per point) × 512 ( ${}^{1}$ H, $F_{1}$ , 13.9 Hz per point) real points (Table S1). Note that since in the NOESY-NOESY-TROSY experiment the data were recorded using the Echo-AntiEcho mode (Kay et al., 1992) only in the $t_{3}$ dimension, immediately preceding acquisition, the *bruk_ranceA.M* macro was not needed after the conversion of the expanded NUS data. The residual in-phase axial peaks along the $F_{2}$ dimension were treated as real peaks and optimally reconstructed by SMILE to suppress the sampling artifacts of the axial signals from spreading to the regions with NOE peaks. The processing macros used for both 4D spectra are included with the raw time-domain data (see Data availability).

### 2.3 Spectrum analysis

Spectra were processed using NMRPipe software (Delaglio et al., 1995); peak picking and spectrum analysis was performed using SPARKY software (Goddard and Kneller, 2008; Lee et al., 2015) as well as NMRDraw (Delaglio et al., 1995). Programs for visualization and analysis were written using freely available python libraries (Hunter, 2007; Harris et al., 2020) as well as NMR-specific python libraries (Helmus and Jaroniec, 2013).

## 3 Results and discussion

Two types of complementary 4D NOE experiments were recorded: (1) 4D TROSY-NOESY-TROSY and (2) 4D NOESY-NOESY-TROSY (Fig. 1). While the former is very similar to the HMQC-NOESY-TROSY experiment used recently for a single α -helical domain with a long rotational correlation time (Barnes et al., 2019), the 4D NOESY-NOESY-TROSY experiment extends earlier work by Kaptein and co-workers (Boelens et al., 1989; Breg et al., 1990).

### 3.1 Recording and analysis of the 4D TROSY-NOESY-TROSY spectrum

The rotational correlation time of the C145A variant of M ${}^{\mathrm{pro}}$ (M ${}_{C145A}^{\mathrm{pro}}$ ) at 25 ${}^{\circ }$ C is ca. 27 ns, and consequently, transverse relaxation is rapid for both ${}^{15}$ N and ${}^{1}$ H ${}^{N}$ nuclei. For this reason, it proved beneficial to substitute a TROSY element for the HMQC or HSQC segment that was previously used for such measurements (Kay et al., 1990; Barnes et al., 2019). Even though the TROSY element only utilizes half of the amide ${}^{1}$ H ${}^{N}$ magnetization present at the start of the pulse sequence, combining its ${}^{15}$ N evolution with sensitivity-enhanced gradient selection during the subsequent $t_{2}$ evolution period (Fig. 1a) limits the loss to $\sqrt{2}$ or even somewhat less when taking the gain from the ${}^{15}$ N Boltzmann magnetization into account (Pervushin et al., 1998). A 2D TROSY spectrum (Fig. S1) of this sample allowed identification of 261 backbone amide peaks out of 293 non-proline residues, suggesting the feasibility of implementing the TROSY version of the 4D NOESY experiment. Conformational exchange on a timescale that results in extensive line broadening and incomplete back-exchange of amides when the protein was purified in ${}^{1}$ H ${}_{2}$ O are the primary causes of the absence of the ca. 30 amide signals.

**Figure 2:**
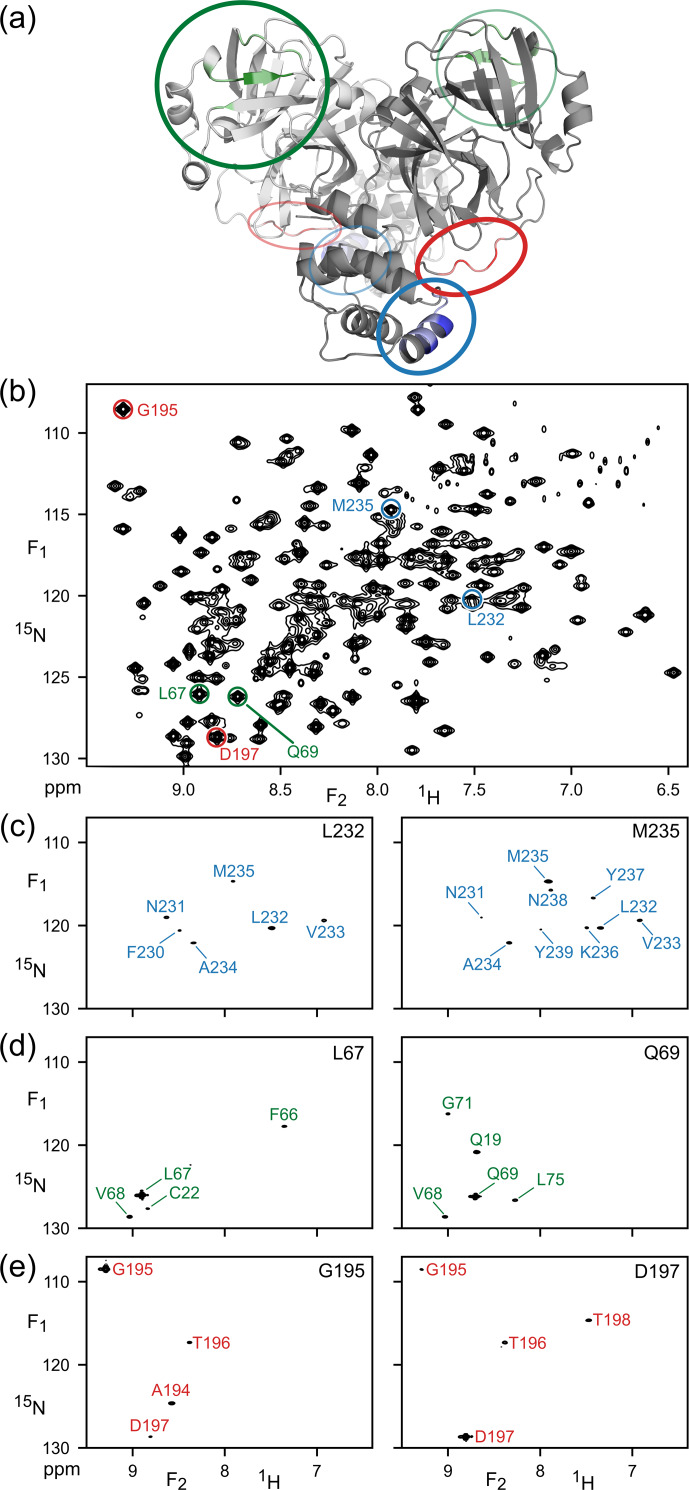
Illustration of amide–amide NOEs in perdeuterated, amide-protonated SARS-CoV-2 Main Protease, observed by 4D TROSY-NOESY-TROSY. **(a)** Ribbon diagram depicting the backbone homodimeric X-ray structure (PDB: 6LU7), with colors marking the regions that are highlighted in ( $F_{1}$ , $F_{2}$ ) cross sections taken at the ( $F_{3}$ , $F_{4}$ ) coordinates of **(c)** L232 and M235 (blue), **(d)** L67 and Q69 (green) and **(e)** G195 and D197 (red). These resonances are marked in **(b)**, which represents the most crowded region of the 800 MHz ${}^{1}$ H– ${}^{15}$ N TROSY-HSQC (the full spectrum is shown in Fig. S1).

The high quality and S/N of the TROSY-HSQC spectrum (Fig. 2b) suggested the feasibility of implementing the TROSY version of the 4D NOESY experiment. Combined with the enhanced relaxation properties during $t_{1}$ and $t_{2}$ evolution of the TROSY-selected coherence, we found experimentally that spectral quality attainable for M ${}_{C145A}^{\mathrm{pro}}$ with the 4D TROSY-NOESY-TROSY was better than with the HMQC-NOESY-TROSY version of the experiment, consistent with the previous report that the TROSY implementation improved both the sensitivity and resolution over the 4D HSQC-NOESY-HSQC (Xia et al., 2000). Figure 2 shows expanded regions of six ( $F_{1}$ , $F_{2}$ ) cross sections through the 4D spectrum, each orthogonal to the ( $F_{3}$ , $F_{4}$ ) frequencies of the six amide correlations that are highlighted in a section of the regular 2D ${}^{1}$ H– ${}^{15}$ N TROSY-HSQC spectrum of Fig. 2b. A total of 231 peaks, out of the 261 peaks in the 2D TROSY spectrum, can be detected as (semi-)resolvable diagonal peaks in the projected ${}^{15}$ N– ${}^{1}$ H ( $F_{3}$ , $F_{4}$ ) plane (data not shown). These numbers do not include the doubling of resonances associated with isomerization of P184.

The cross sections exemplify the power of 4D analysis for three types of secondary structure: α -helix (Fig. 2c), β -sheet (Fig. 2d), and a loop region (Fig. 2e). Due to the long NOE mixing time used in this experiment (200 ms), substantial spin diffusion occurs, which results in numerous NOE correlations for each amide. For example, α -helical residues L232 and M235 not only show NOE interactions with one another, but also share NOE cross peaks to V233 and A234, with M235 even showing a weak cross peak to N231. Such correlations are particularly useful for validating the assignments obtained from the limited number of triple-resonance backbone assignment experiments that are applicable to larger proteins such as M ${}^{\mathrm{pro}}$ .

The amides of L67 and Q69 in strand β 4 only share a single NOE, to sequential residue V68, but they show valuable long-range NOEs to amide protons in strands β 1 (C22) and β 5 (L75). G195 and D197, located in the long loop that connects strand β 13 to helix α 6, have an NOE to one another as well as sequential NOEs but show no long-range interactions, consistent with the X-ray structure (Douangamath et al., 2020). However, NOEs from L67 or Q69 to T21 or Q19 are not observed, despite close proximity, due to the minimal back-exchange of amide protons in the β 1 strand.

It is interesting to compare the diagonal peak intensities in these various cross sections of the TROSY-NOESY-TROSY spectrum. Diagonal intensity is a function of the amount of amide ${}^{1}$ H z magnetization present at the start of the pulse sequence, i.e., it depends on the non-selective longitudinal relaxation time of the amide proton, but also on the attenuation of this magnetization during the NOE mixing time, in other words, on the selective longitudinal relaxation time which is dominated by J(0) spectral density terms. The latter dominate the differences in diagonal intensity seen in the various cross sections. For example, the helical amides of L232 and M235 rapidly lose their magnetization to their proximate sequential amide neighbors, separated by ca. 2.7 Å, that each are in close contact with other neighboring protons. By contrast, none of the L67, Q69, G195 and D197 amides are closer than 3.7 Å from any neighboring protonated amide in the 1.25 Å X-ray structure of M ${}^{\mathrm{pro}}$ (Douangamath et al., 2020), causing their diagonal intensities to remain high.

### 3.2 Recording and analysis of the 4D NOESY-NOESY-TROSY spectrum

As highlighted by the work of Kaptein and co-workers, 3D NOE-NOE experiments provided an effective method for studying the ${}^{1}$ H– ${}^{1}$ H cross-relaxation network in proteins in more detail. Here, we extend this powerful experiment to four dimensions, making it more straightforward to analyze such a spectrum while limiting the relaxation pathways by perdeuteration of the protein.

The pulse scheme of this 4D NOESY-NOESY-TROSY is shown in Fig. 1b. It represents a straightforward extension of the original NOE-NOE 3D experiment (Boelens et al., 1989) but with the detection period substituted by the gradient-enhanced 2D ${}^{1}$ H– ${}^{15}$ N TROSY scheme (Pervushin et al., 1998). The latter enhances the attainable spectral resolution in the $t_{3}$ and $t_{4}$ dimensions, while dispersing the detected ${}^{1}$ H ${}^{N}$ resonances in the ${}^{15}$ N dimension. A number of minor technical considerations are also relevant in this respect. (1) First, in order to maximize the number of ( $t_{1}$ , $t_{2}$ , $t_{3}$ ) data points sampled, the phase cycling of the 4D experiment was reduced to four steps, and the observed spectral window was restricted to the region downfield of the H ${}_{2}$ O resonance. To prevent bleeding in of several weaker imperfectly deuterated aliphatic or exchangeable resonances present in the upfield spectral region, selective-EBURP2 and reverse-EBURP2 pulses (Geen and Freeman, 1991) were used to also restrict the regions where ${}^{1}$ H resonances were excited to those resonating downfield from the water resonance. As a result, no NOE peaks from a few amide protons resonating near water or upfield from water were observed. (2) Recording of a 4D NMR spectrum at adequate resolution requires the use of non-uniform sampling. High quality NUS reconstruction of a 4D NMR spectrum can be accomplished by the SMILE program (Ying et al., 2017) but this as well as most other NUS reconstruction software performs better if the various time domains are acquired in a manner that results in either a 0 ${}^{\circ }$ or a 180 ${}^{\circ }$ linear phase correction across the spectrum. For this purpose, and to ensure that the non-suppressed axial peaks can be optimally reconstructed, which requires 0 ${}^{\circ }$ linear phase correction, it was preferable to insert a non-selective 90 ${}^{\circ }$ ${}_{x}$ 207 ${}^{\circ }$ ${}_{y}$ 90 ${}^{\circ }$ ${}_{x}$ composite ${}^{1}$ H inversion pulse (highlighted as the green open bar in Fig. 1b), followed by a second such pulse that reverses any phase imperfections introduced by the first composite pulse (Hwang et al., 1997). Specifically, the $\varphi_{1}$ phase cycling serves to eliminate axial peaks in the $t_{1}$ dimension caused by pulse imperfection as well as $T_{1}$ relaxation and amide exchange with solvent during $T_{m1}$ , while also suppressing axial peaks in the $t_{2}$ dimension resulting from $T_{1}$ relaxation and water exchange during $T_{m2}$ . To minimize the number of phase cycling steps, $\varphi_{2}$ was not phase cycled. However, this resulted in small residual axial peaks along the $F_{2}$ dimension caused by pulse imperfections. To ensure that these residual axial peaks were absorptive in the final spectrum, thereby simplifying SMILE NUS reconstruction, an echo is generated by the application of two composite ${}^{1}$ H 180 ${}^{\circ }$ pulses in order to suppress initial chemical shift evolution at $t_{2}=0$ , thereby eliminating the need for a linear phase correction. Considering that the real and imaginary components of the residual axial signals have the same amplitude, they result in a 45 ${}^{\circ }$ phase error for the axial peaks in the $F_{2}$ dimension. Shifting the $\varphi_{2}$ phase by - 45 ${}^{\circ }$ ensures that the NOE and axial peaks both can be phased absorptive using the same phase correction, thus facilitating NUS processing.

**Figure 3:**
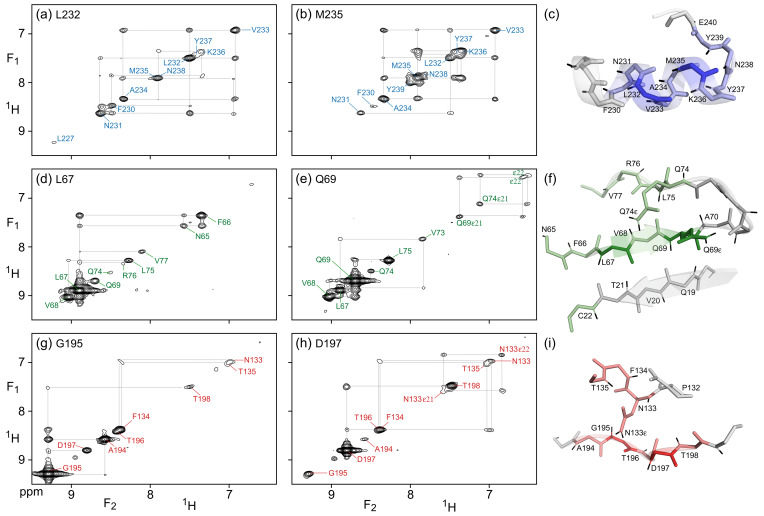
( $F_{1}$ , $F_{2}$ ) cross sections through the 4D NOESY-NOESY-TROSY spectrum of M ${}_{C145A}^{\mathrm{pro}}$ , taken orthogonal to the ( $F_{3}$ , $F_{4}$ ) TROSY-HSQC plane at the ${}^{15}$ N, ${}^{1}$ H frequencies of **(a)** L232, **(b)** M235, **(d)** L67, **(e)** Q69), **(g)** G195, and **(h)** D197. These cross sections show diagonal resonances for amide protons that are within long-range contact of the selected amide, either through direct or indirect NOE transfer during the two mixing periods that have a total duration of 50 + 300 ms. Off-diagonal resonances in these cross sections correspond to NOE magnetization transfer during the 50 ms mixing period that separates the $t_{1}$ and $t_{2}$ evolution periods. Colors match those of the corresponding residues in Fig. 2. Expanded views of the structural elements (PDB: 6LU7) that gave rise to the observed NOEs are shown in panels **(c)**, **(f)**, and **(i)**.

Compared to the 4D TROSY-NOESY-TROSY pulse scheme, the 4D NOESY-NOESY-TROSY experiment avoids the lossy magnetization transfer step from ${}^{1}$ H to ${}^{15}$ N and back (leading to a slightly larger number of 241 diagonal peaks on the ${}^{15}$ N– ${}^{1}$ H ( $F_{3}$ , $F_{4}$ ) projected plane, compared to 231 for TROSY-NOESY-TROSY). Instead, its magnetization is simply transferred, in part, to its nearest neighbors by cross-relaxation during the first NOE mixing period of duration $T_{m1}=50$ ms. There is virtually no loss in total spin polarization summed over the initial “starting spin”, whose $t_{1}$ evolution is monitored, and those of its immediate neighbors that are within cross-relaxation contact. As a result, the intrinsic sensitivity of such NOESY-NOESY-TROSY measurements is quite high, allowing the choice of a long duration of 300 ms for the second NOE mixing time, $T_{m2}$ . During this second, much longer mixing time, the z magnetization distributes over considerable distances due to indirect transfers (Fig. 3). Even in this extensively perdeuterated protein, NOEs to nearly a dozen neighboring protons are observed on the diagonals of the ( $F_{1}$ , $F_{2}$ ) cross sections, taken at the same ( ${}^{15}$ N, ${}^{1}$ H) frequencies used for illustrating the utility of the 4D TROSY-NOESY-TROSY spectrum of Fig. 2. However, as pointed out by Boelens et al. (1989) and Breg et al. (1990), the NOE-NOE combination offers a wealth of new information on the cross-relaxation pathways that led to the long-distance NOEs, substantially aiding both the assignment and analysis of distance information. Below, we briefly highlight a few examples.

As expected, α -helical residue L232 shows intense cross peaks to both of its sequential neighbors, N231 and V233, as well as a weaker cross peak to F230. Despite the relatively short mixing time of only 50 ms that separates $t_{1}$ and $t_{2}$ evolution, the latter must result mostly from indirect transfer through N231, because N231 and F230 share an intense cross peak. So in effect, each cross section through the 4D spectrum shown in Fig. 3 corresponds to a 2D NOESY spectrum of a small, localized, region within the protein structure – making its analysis far simpler. For residues with few neighbors, direct NOE contacts between neighbors separated by as much as 4.5 Å give rise to quite intense cross peaks after 50 ms NOE mixing, as exemplified by the contacts between G195 and its A194 and T196 neighbors (Fig. 3g). A weaker cross peak between G195 and D197, at an interproton distance of 6.4 Å, appears not to be mediated by spin diffusion because the G195 and D197 panels (Fig. 3g) show no common strong NOE to any visible resonance. However, the possibility that the hydroxyl proton of T196 serves as a relay partner cannot be excluded.

The NOESY-NOESY-TROSY spectrum also shows multiple NOEs to sidechain amide protons that are not visible in the TROSY-NOESY-TROSY spectrum because the TROSY element does not select magnetization transfer for NH ${}_{2}$ groups. For example, D197 shows long-range NOEs to the N133 carboxamide protons, whereas Q69 shows NOEs to both its own carboxamide protons and to those of Q74. The non-equivalent NH ${}_{2}$ pairs are readily recognized by cross peak to diagonal peak intensity ratios that are close to one, owing to their short interproton distance.

## 4 Concluding remarks

The spectra shown in this study were recorded during the summer of 2020, when access to campus facilities was strongly restricted due to COVID-19 pandemic mitigation efforts. These restrictions allowed for much lengthier acquisition of spectra than commonly used, for a total of 8 d for the two 4D spectra. As a benefit of NUS reconstruction, it is possible to generate spectra of the same resolution recorded in any fraction of that time. Alternatively, we can discard the data recorded at the longest values of $t_{1}$ , $t_{2}$ , and $t_{3}$ . Indeed, processing the same time-domain data sets but shortening the time domains using a previously described protocol that considers the total normalized length of the 3D ( $t_{1}$ , $t_{2}$ , $t_{3}$ ) time-domain vector (Ying et al., 2019), using only one-third of the acquired time-domain data yields spectra that are very similar to the ones shown in Figs. 2 and 3, albeit at slightly lower resolution and signal to noise, due to the use of 3-fold less time-domain data. Nevertheless, the quality of the resulting spectra remains excellent, with near-identical information content (Figs. S2 and S3 in the Supplement).

Use of the lengthy data acquisition times needed to collect the 4D spectra requires a high stability sample, which in our case benefited from the C145A active site mutation, protecting the sample from auto-proteolysis. As with all NMR experiments, S/N is approximately proportional to sample concentration. Therefore, working at high concentrations benefits S/N of these experiments that involve multiple magnetization transfer steps, an issue that is particularly important for NOE experiments where magnetization from a single nucleus is distributed over many neighbors.

We note that the TROSY-NOESY-TROSY experiment used a long NOE mixing time of 200 ms, such as to increase the number of observed connectivities by adding indirect NOE effects, including spin diffusion through hydroxyl protons (Koharudin et al., 2003), thereby aiding the assignment process. The use of a 50 ms NOE mixing period in the subsequent 4D NOESY-NOESY-TROSY experiment then provided a semi-quantitative measure of distance between these protons and their neighbors. Indeed, as pointed out by Kaptein and co-workers, recording of NOE-NOE spectra provides important experimental data on the pathway of magnetization transfer during NOE mixing. Such information could be used to convert these data into more quantitative distance information than the typical qualitative analysis of NOE intensities, potentially leading to the generation of higher resolution structures (Vogeli et al., 2009, 2012). Quantitative NOE interpretation traditionally relied on the recording of a series of NOE buildup data, which can become comparably time-consuming as the recording of 4D NMR spectra if resonance overlap is a limiting factor, as typically is the case for NOE spectra. This problem is further exacerbated by the spectral crowding of large proteins, particularly in the ${}^{1}$ H dimension, and while 3D spectra may give higher signal-to-noise ratios than 4D spectra, downstream analysis frequently requires extensive disambiguation of overlapped peaks. Our study of M ${}_{C145A}^{\mathrm{pro}}$ shows that a large number of semi-quantitative NOE distances become accessible by recording of 4D NMR spectra on a perdeuterated larger protein with little or no ambiguity about the nuclei involved.

While the high signal to noise and spectral simplicity of working with perdeuterated proteins has long been recognized (Torchia et al., 1988; Lemaster and Richards, 1988; Grzesiek et al., 1993; Tugarinov et al., 2004) the number of structural restraints accessible used to be small. Our present study demonstrates that a much larger number of NOE interactions becomes available by the recording of 4D NOE spectra. Moreover, it highlights the exquisite detail and value of NOE-NOE interaction analysis explored by the Kaptein group and it demonstrates that this approach is highly suitable for the larger biomolecules and biomolecular complexes being explored today, in particular when using extensive perdeuteration. Therefore, we believe that the recording of high quality 4D NMR spectra of the type presented in this study is entirely practical and invaluable for the structural and functional analysis of large proteins and their complexes, with possible extension to the study of nucleic acids. We note, however, that in the absence of extensive deuteration the dilution of nuclear magnetization over sidechain resonances will strongly lower the sensitivity of the experiment, which is further exacerbated by decreased effectiveness of TROSY-based line narrowing in such samples. On the other hand, adaptations of the NOESY-NOESY-TROSY experiment to methyl-protonated but otherwise perdeuterated proteins (Tugarinov et al., 2005b) are expected to be readily feasible.

## Supplement

10.5194/mr-2-129-2021-supplement The supplement related to this article is available online at: https://doi.org/10.5194/mr-2-129-2021-supplement.

## Data Availability

The raw Bruker NMR data sets including the acquisition parameters and NUS sampling lists, pulse programs, include file, and NMRPipe processing scripts are available for download from Zenodo: https://zenodo.org/record/4625615 (Robertson et al., 2021).

## References

[bib1.bib1] Altincekic N Large-scale recombinant production of the SARS-CoV-2
proteome for high-throughput and structural biology applications. Frontiers
in Molecular Biosciences.

[bib1.bib2] Barnes CA, Shen Y, Ying JF, Takagi Y, Torchia DA, Sellers JR, Bax A (2019). Remarkable Rigidity of the Single alpha-Helical Domain of Myosin-VI As Revealed by NMR Spectroscopy. J Am Chem Soc.

[bib1.bib3] Boelens R, Koning TMG, Kaptein R (1988). Determination of Biomolecular Structures From Proton-Proton Noes Using a Relaxation Matrix Approach. J Mol Struct.

[bib1.bib4] Boelens R, Vuister GW, Koning TMG, Kaptein R (1989). Observation of spin diffusion in biomolecules by 3-dimensional NOE-NOE spectroscopy. J Am Chem Soc.

[bib1.bib5] Boras B, Jones RM, Anson BJ, Arenson D, Aschenbrenner L, Bakowski MA, Beutler N, Binder J, Chen E, Eng H, Hammond H, Hammond J, Haupt RE, Hoffman R, Kadar EP, Kania R, Kimoto E, Kirkpatrick MG, Lanyon L, Lendy EK, Lillis JR, Logue J, Luthra SA, Ma C, Mason SW, McGrath ME, Noell S, Obach RS, O'Brien MN, O'Connor R, Ogilvie K, Owen D, Pettersson M, Reese MR, Rogers TF, Rossulek MI, Sathish JG, Shirai N, Steppan C, Ticehurst M, Updyke LW, Weston S, Zhu Y, Wang J, Chatterjee AK, Mesecar AD, Frieman MB, Anderson AS, Allerton C (2021). Discovery of a Novel Inhibitor of Coronavirus 3CL Protease for the Potential Treatment of COVID-19. bioRxiv.

[bib1.bib6] Breg JN, Boelens R, Vuister GW, Kaptein R (1990). 3D NOE-NOE spectroscopy of proteins – Observation of sequential 3D NOE cross peaks in Arc repressor. J Magn Reson.

[bib1.bib7] Clore GM, Kay LE, Bax A, Gronenborn AM (1991). Four-dimensional 13C/13C-edited nuclear Overhausesr enhancement spectroscopy of a protein in solution: Application to interleukin 1
β. Biochemistry.

[bib1.bib8] Delaglio F, Grzesiek S, Vuister GW, Zhu G, Pfeifer J, Bax A (1995). NMRpipe - a multidimensional spectral processing system based on Unix pipes. J Biomol NMR.

[bib1.bib9] Diercks T, Truffault V, Coles M, Millett O (2010). Diagonal-Free 3D/4D HN, HN-TROSY-NOESY-TROSY. J Am Chem Soc.

[bib1.bib10] Douangamath A, Fearon D, Gehrtz P, Krojer T, Lukacik P, Owen CD, Resnick E, Strain-Damerell C, Aimon A, Abranyi-Balogh P, Brandao-Neto J, Carbery A, Davison G, Dias A, Downes TD, Dunnett L, Fairhead M, Firth JD, Jones SP, Keeley A, Keseru GM, Klein HF, Martin MP, Noble MEM, O'Brien P, Powell A, Reddi RN, Skyner R, Snee M, Waring MJ, Wild C, London N, von Delft F, Walsh MA (2020). Crystallographic and electrophilic fragment screening of the SARS-CoV-2 main protease. Nat Commun.

[bib1.bib11] Geen H, Freeman R (1991). Band-selective radiofrequency pulses. J Magn Reson.

[bib1.bib12] Goddard TD, Kneller DG (2008). Sparky 3.

[bib1.bib13] Grzesiek S, Bax A (1993). The Importance of Not Saturating H
${}_{2}$
O in Protein NMR. Application to Sensitivity Enhancement and NOE Measurement. J Am Chem Soc.

[bib1.bib14] Grzesiek S, Anglister J, Ren H, Bax A (1993). ${}^{13}$
C line narrowing by 
${}^{2}$
H decoupling in 
${}^{2}$
H/
${}^{13}$
C/
${}^{15}$
N-enriched Proteins. Application to triple resonance 4D connectivity of sequential amides. J Am Chem Soc.

[bib1.bib15] Grzesiek S, Wingfield P, Stahl S, Kaufman JD, Bax A (1995). Four-dimensional 
${}^{15}$
N-separated NOESY of slowly tumbling perdeuterated 
${}^{15}$
N-enriched proteins. Applications to HIV-1 Nef. J Am Chem Soc.

[bib1.bib16] Harris CR, Millman KJ, van der Walt SJ, Gommers R, Virtanen P, Cournapeau D, Wieser E, Taylor J, Berg S, Smith NJ, Kern R, Picus M, Hoyer S, van Kerkwijk MH, Brett M, Haldane A, del Rio JF, Wiebe M, Peterson P, Gerard-Marchant P, Sheppard K, Reddy T, Weckesser W, Abbasi H, Gohlke C, Oliphant TE (2020). Array programming with NumPy. Nature.

[bib1.bib17] Helmus JJ, Jaroniec CP (2013). Nmrglue: an open source Python package for the analysis of multidimensional NMR data. J Biomol NMR.

[bib1.bib18] Hunter JD (2007). Matplotlib: A 2D graphics environment. Comput Sci Eng.

[bib1.bib19] Hwang TL, van Zijl PCM, Garwood M (1997). Broadband adiabatic refocusing without phase distortion. J Magn Reson.

[bib1.bib20] Ikura M, Kay LE, Bax A (1990). A novel approach for sequential assignment of 
${}^{1}$
H, 
${}^{13}$
C, and 
${}^{15}$
N spectra of larger proteins: heteronuclear triple-resonance three-dimensional NMR spectroscopy. application to calmodulin. Biochemistry.

[bib1.bib21] Kay LE, Clore GM, Bax A, Gronenborn AM (1990). Four-dimensional heteronuclear triple-resonance NMR spectroscopy of interleukin-1B in solution. Science.

[bib1.bib22] Kay LE, Keifer P, Saarinen T (1992). Pure Absorption Gradient Enhanced Heteronuclear Single Quantum Correlation Spectroscopy with Improved Sensitivity. J Am Chem Soc.

[bib1.bib23] Koharudin LMI, Bonvin A, Kaptein R, Boelens R (2003). Use of very long-distance NOEs in a fully deuterated protein: an approach for rapid protein fold determination. J Magn Reson.

[bib1.bib24] Lee W, Tonelli M, Markley JL (2015). NMRFAM-SPARKY: enhanced software for biomolecular NMR spectroscopy. Bioinformatics.

[bib1.bib25] Lemaster DM, Richards FM (1988). NMR sequential assignment of Escherichia-coli thioredoxin utilizing random fractional deuteration. Biochemistry.

[bib1.bib26] Marion D, Driscoll PC, Kay LE, Wingfield PT, Bax A, Gronenborn AM, Clore GM (1989). Overcoming the overlap problem in the assignment of 
${}^{1}$
H NMR spectra of larger proteins by use of three-dimensional heteronuclear 
${}^{1}$
H-
${}^{15}$
N Hartmann-Hahn-multiple quantum coherence and nuclear Overhauser-multiple quantum coherence spectroscopy: application to interleukin 1
β. Biochemistry.

[bib1.bib27] Marion D, Kay LE, Sparks SW, Torchia DA, Bax A (1989). Three-dimensional heteronuclear NMR of 
${}^{15}$
N-labeled proteins. J Am Chem Soc.

[bib1.bib28] Oschkinat H, Griesinger C, Kraulis PJ, Sorensen OW, Ernst RR, Gronenborn AM, Clore GM (1988). 3-Dimensional NMR spectroscopy of a protein in solution. Nature.

[bib1.bib29] Pervushin K, Riek R, Wider G, Wuthrich K (1997). Attenuated T2 relaxation by mutual cancellation of dipole-dipole coupling and chemical shift anisotropy indicates an avenue to NMR structures of very large biological macromolecules in solution. Proc Natl Acad Sci USA.

[bib1.bib30] Pervushin KV, Wider G, Wuthrich K (1998). Single transition-to-single transition polarization transfer (ST2-PT) in [N15,H1]-TROSY. J Biomol NMR.

[bib1.bib31] Robertson A, Ying J, Bax A Zenodo.

[bib1.bib32] Rovnyak D, Frueh DP, Sastry M, Sun ZYJ, Stern AS, Hoch JC, Wagner G (2004). Accelerated acquisition of high resolution triple-resonance spectra using non-uniform sampling and maximum entropy reconstruction. J Magn Reson.

[bib1.bib33] Torchia DA, Sparks SW, Bax A (1988). Delineation of Alpha-Helical Domains in Deuteriated Staphylococcal Nuclease By 2d Noe Nmr-Spectroscopy. J Am Chem Soc.

[bib1.bib34] Tugarinov V, Muhandiram R, Ayed A, Kay LE (2002). Four-dimensional NMR spectroscopy of a 723-residue protein: Chemical shift assignments and secondary structure of malate synthase G. J Am Chem Soc.

[bib1.bib35] Tugarinov V, Hwang PM, Kay LE (2004). Nuclear magnetic resonance spectroscopy of high-molecular-weight proteins. Annu Rev Biochem.

[bib1.bib36] Tugarinov V, Choy WY, Orekhov VY, Kay LE (2005). Solution NMR-derived global fold of a monomeric 82-kDa enzyme. Proc Natl Acad Sci USA.

[bib1.bib37] Tugarinov V, Kay LE, Ibraghimov I, Orekhov VY (2005). High-resolution four-dimensional H-1-C-13 NOE spectroscopy using methyl-TROSY, sparse data acquisition, and multidimensional decomposition. J Am Chem Soc.

[bib1.bib38] Vogeli B, Segawa TF, Leitz D, Sobol A, Choutko A, Trzesniak D, van Gunsteren W, Riek R (2009). Exact Distances and Internal Dynamics of Perdeuterated Ubiquitin from NOE Buildups. J Am Chem Soc.

[bib1.bib39] Vogeli B, Kazemi S, Guntert P, Riek R (2012). Spatial elucidation of motion in proteins by ensemble-based structure calculation using exact NOEs. Nat Struct Mol Biol.

[bib1.bib40] Vuister GW, Boelens R, Kaptein R (1988). Non-selective 3-dimensional NMR spectroscopy – The 3D NOE-HOHAHA experiment. J Magn Reson.

[bib1.bib41] Wagner G (1993). Prospects for NMR of large proteins. J Biomol NMR.

[bib1.bib42] Wüthrich K (1986). NMR of Proteins and Nucleic Acids.

[bib1.bib43] Xia YL, Sze KH, Zhu G (2000). Transverse relaxation optimized 3D and 4D N-15/N-15 separated NOESY experiments of N-15 labeled proteins. J Biomol NMR.

[bib1.bib44] Ying J, Delaglio F, Torchia DA, Bax A (2017). Sparse multidimensional iterative lineshape-enhanced (SMILE) reconstruction of both non-uniformly sampled and conventional NMR data. J Biomol NMR.

[bib1.bib45] Ying JF, Barnes CA, Louis JM, Bax A (2019). Importance of time-ordered non-uniform sampling of multidimensional NMR spectra of A beta(1-42) peptide under aggregating conditions. J Biomol NMR.

[bib1.bib46] Zuiderweg ERP, Fesik SW (1989). Heteronuclear Three-Dimensional NMR Spectroscopy of the Inflammatory Protein C5
${}_{a}$. Biochemistry.

[bib1.bib47] Zuiderweg ERP, Petros AM, Fesik SW, Olejniczak ET (1991). 4-Dimensional [C-13, H-1, C-13, H-1] Hmqc-Noe-Hmqc Nmr-Spectroscopy – Resolving Tertiary Noe Distance Constraints in the Spectra of Larger Proteins. J Am Chem Soc.

