# Supplementary material for: Four-dimensional NOE-NOE spectroscopy of SARS-CoV-2 Main Protease to facilitate resonance assignment and structural analysis
Source: Magn Reson (Gott). 2021 Apr 13;2(1):129–38. doi: 10.5194/mr-2-129-2021 (PMC10539749; doi:10.5194/mr-2-129-2021)
Supplement: The supplement related to this article is available online at: https://doi.org/10.5194/mr-2-129-2021-supplement. [file mr-2-129-supplement.pdf]

Supplement of Magn. Reson., 2, 129–138, 2021
https://doi.org/10.5194/mr-2-129-2021-supplement
© Author(s) 2021. CC BY 4.0 License.

Open Access
MAGNETIC RESONANCE

Supplement of

Four-dimensional NOE-NOE spectroscopy of SARS-CoV-2 Main Protease to facilitate resonance assignment and structural analysis

Angus J. Robertson et al.

Correspondence to: Ad Bax (bax@nih.gov)

The copyright of individual parts of the supplement might differ from the article licence.

Additional experimental information

Overnight starter cultures of 10 mL with increasing (0%, 90%, 99.8%) ^2H_2O content were performed (37°C, 200 rpm) to acclimatize the BL21(DE3) cells containing the $M^{pro}{}_{C145A}$ plasmid to 98.8% ^2H_2O M9 media, with a *ca.* 50x dilution of starter cultures between steps. The M9 minimal medium (Sambrook et al. 1989) for starter- (and large-) culture was supplemented with (per liter of culture); 1 mL $MgCl_2$ (1M), 100uL $CaCl_2$ (1M), 650 mL trace elements (as in (Reed et al. 2003)), 50 mg/L Kanamycin Sulfate, 1g ^{15}N-NH_4Cl (Cambridge Isotopes) - with 2g ^2H-Glucose (97% ^2H; Cambridge Isotopes) and 1g $^2H^{15}N$-Isogro (Isotec) as the sole carbon sources. The full 10 mL starter-culture was added to 500 mL 98.8% ^2H_2O M9 growth media and grown at 37°C, 200 rpm, and cells were induced with 1 mM IPTG when $A_{600} = 0.8$ for 4h at 37°C, 200 rpm before being harvested by centrifugation and frozen at -80°C overnight. All reagents for the recombinant-protein overexpression step were prepared in 99.8% ^2H_2O and filter-sterilized (Millipore Express® PES membrane), which combined with >97% deuteration of carbon sources resulted in a >98% perdeuteration of the protein.

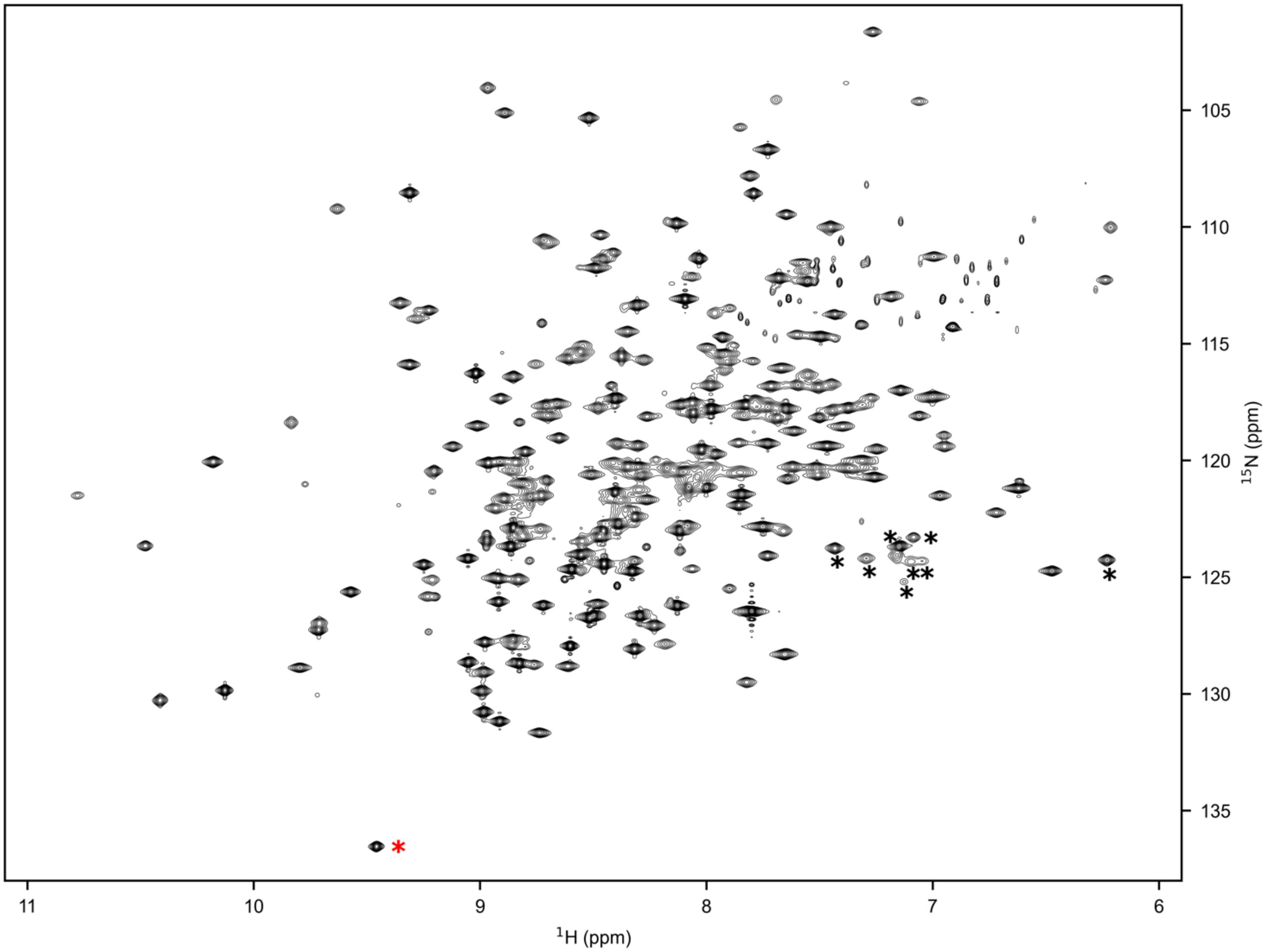

Figure S1. ^{1}H-^{15}N-TROSY-HSQC spectrum of 1.8 mM (0.9 mM dimer) ^{2}H/^{15}N labeled M^{pro}_{C145A} as described in the main text was recorded at 25 ˚C at 800 MHz (1142* and 150* complex data points corresponding to 79 ms for the ^{1}H and 48 ms for the ^{15}N dimensions, respectively, interscan delay 3.5 s). A total of 261 non-proline backbone amide peaks are present in the spectrum (some substantially overlapped). For acquisition of the 4D spectra, the ^{15}N spectral width was reduced and the most downfield shifted peak (indicated with a red asterisk) was aliased to the upfield end of the spectrum. Aliased arginine sidechain resonances are marked with black asterisks.

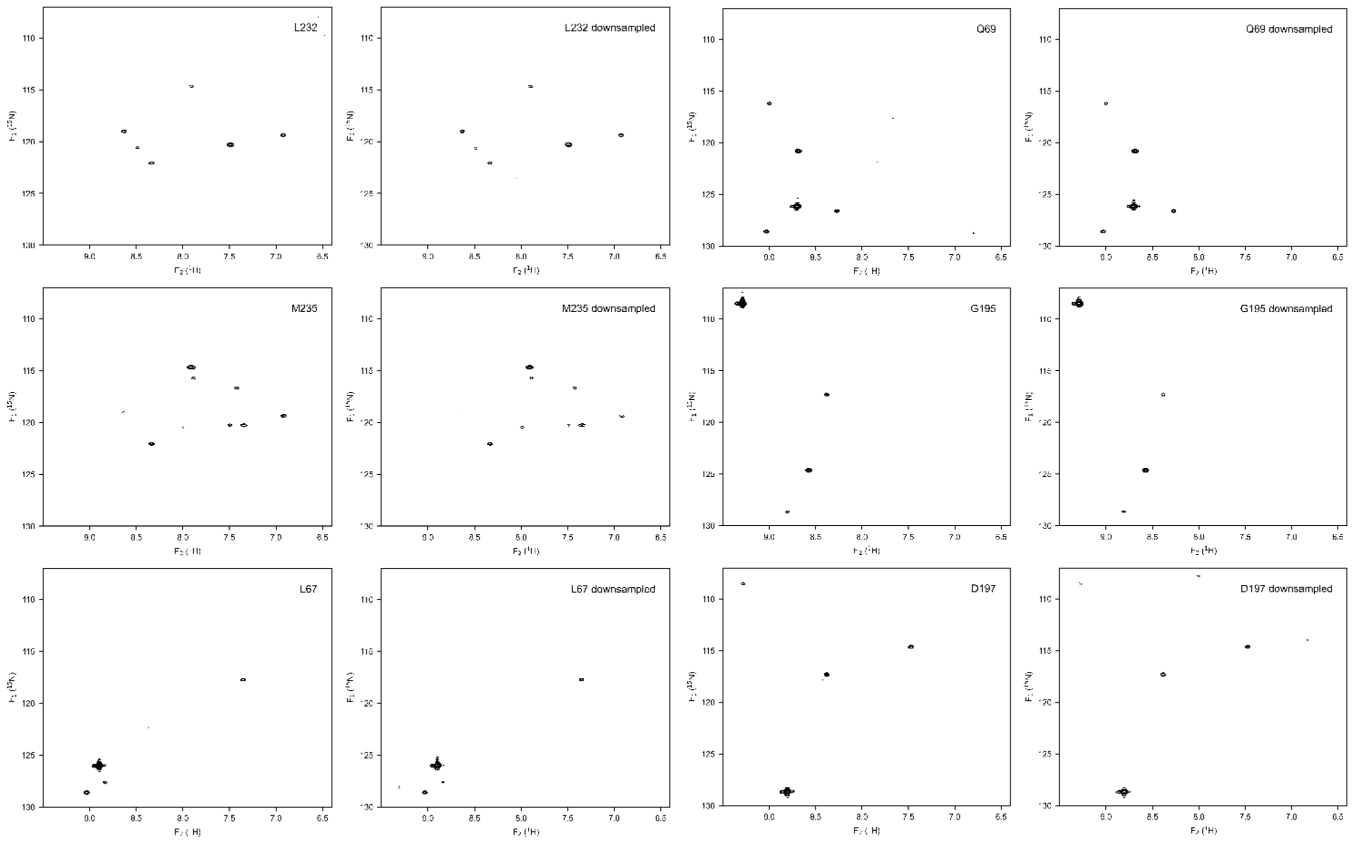

Figure S2. Comparison of the cross sections through the 4D TROSY-NOESY-TROSY spectrum shown in Figure 2 of the main text (left) with the corresponding cross sections obtained from a 33% subset of the sampled time domain data (panels labeled "downsampled"), processed identically to the full dataset, and contoured at the same level relative to the noise. The time domain datapoints used for the "downsampled" spectrum were selected by limiting the length of the total time domain vector according to $[(t_1/t_{1,max})^2 + (t_2/t_{2,max})^2 + (t_3/t_{3,max})^2]^{0.5} \leq 0.84$, where $t_{1,max}$, $t_{2,max}$, and $t_{3,max}$ are the length of the time domain in the fully sampled data set.

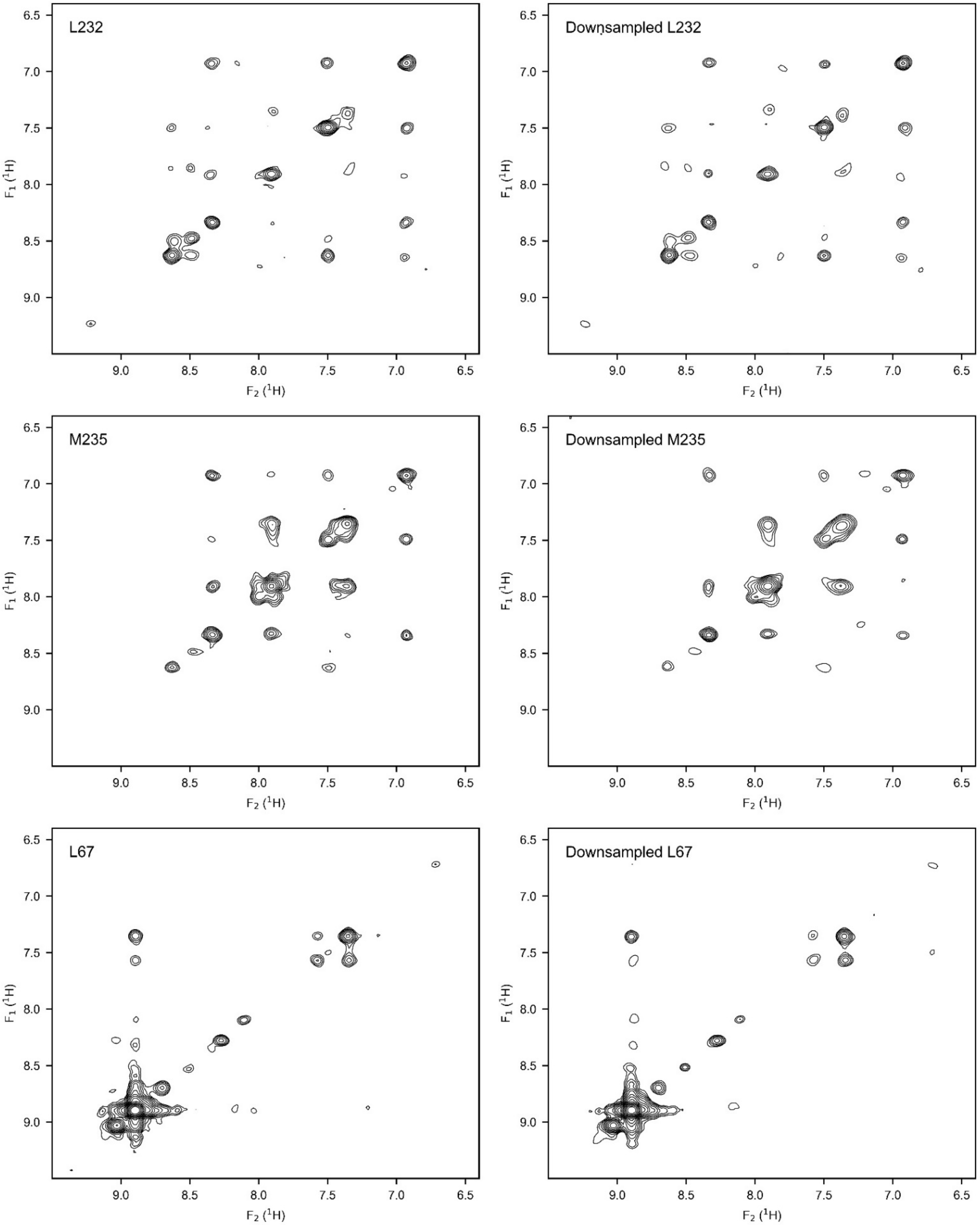
L232
Downsampled L232
M235
Downsampled M235
L67
Downsampled L67
F1 (1H)
F2 (1H)
6.5
7.0
7.5
8.0
8.5
9.0

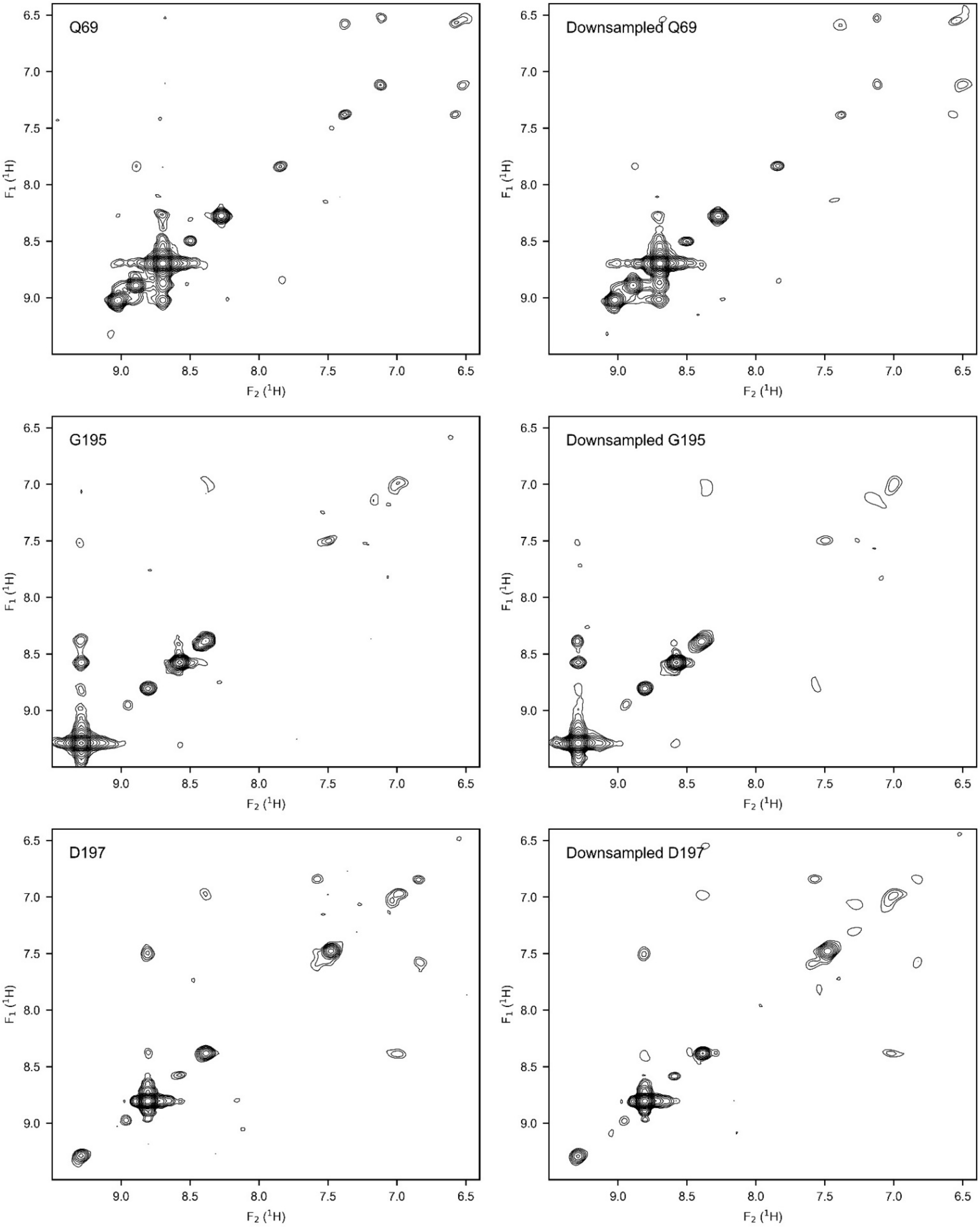
Q69
Downsampled Q69
G195
Downsampled G195
D197
Downsampled D197
F1 (1H)
F2 (1H)
6.5
7.0
7.5
8.0
8.5
9.0
9.0
8.5
8.0
7.5
7.0
6.5

Figure S3. Comparison of the same cross sections through the 4D NOESY-NOESY-TROSY spectrum shown in Figure 3 of the main text with the corresponding cross sections obtained from a 33% subset of the sampled time domain data, processed identically to the full dataset, and contoured at the same level relative to the noise. The time domain datapoints used for the "downsampled" spectrum were selected by limiting the length of the total time domain vector according to $[(t_1/t_{1,max})^2 + (t_2/t_{2,max})^2 + (t_3/t_{3,max})^2]^{0.5} \leq 0.847$, where $t_{1,max}$, $t_{2,max}$, and $t_{3,max}$ are the length of the time domain in the fully sampled data set.

Table S1. Acquisition, NUS sampling, and SMILE reconstruction parameters for the TROSY-NOESY-TROSY and NOESY-NOESY-TROSY experiments.

Experiment	TROSY-NOESY-TROSY				NOESY-NOESY-TROSY			
Dimension	F_4	F_3	F_2	F_1	F_4	F_3	F_2	F_1
Nucleus observed	^1H	^{15}N	^1H	^{15}N	^1H	^{15}N	^1H	^1H
Quadrature detection	Direct	Echo-AntiEcho	States-TPPI	Echo-AntiEcho	Direct	Echo-AntiEcho	States-TPPI	States-TPPI
Topspin TD size	3072	180	182	180	3072	180	120	120
No. of increment (i.e. complex pairs)	1536	90	91	90	1536	90	60	60
Extended No. of increment by SMILE	1536	135	136	135	1536	135	90	90
Spectral width (Hz)	12820.5	2564.1	4545.5	2564.1	16025.6	2564.1	5000.0	5000.0
Acquisition time (msec)	119.8	35.1	20.0	35.1	95.8	35.1	12.0	12.0
SMILE extended acquisition time (msec)	119.8	52.7	29.9	52.7	95.8	52.7	18.0	18.0
Topspin NUS T_2 for exp. weighting (sec)	n/a	1.0	1.0	1.0	n/a	1.0	1.0	1.0
Topspin NUS sampling random seed	n/a	54321			n/a	2005291846		
NUS sampling percentage (effective)	n/a	0.54% (0.16%) [a]			n/a	1.69% (0.50%) [a]		
Total No. of FID recorded	n/a	31896			n/a	43856		
No. of real points in the spectrum	614	512	512	512	492	512	512	512
Spectral digital resolution (Hz/point)	6.3	5.0	8.9	5.0	7.8	4.7	13.9	13.8
No. of threads for SMILE[b]	16				16			
Max allowed memory for SMILE (GB)	64 (61.6)[c]				64 (63.2)[c]			
SMILE reconstruction time (hour(s))[d]	8.5				1.4			

[a] The effective sampling percentage after the default 50% SMILE extension in each of the indirect dimensions
[b] Reconstruction running on a Ubuntu 20.0 workstation with 32 Intel Xeon Gold 6226R CPUs (2.90 GHz) and 512 GB memory available
[c] The actual amount of memory allocated by SMILE
[d] The wall time for the SMILE reconstruction only, not including the data conversion, expansion and post reconstruction conventional NMRPipe processing time

References

Reed, M. A. C., A. M. Hounslow, K. H. Sze, I. G. Barsukov, L. L. P. Hosszu, A. R. Clarke, C. J. Craven and J. P. Waltho (2003). "Effects of Domain Dissection on the Folding and Stability of the 43 kDa Protein PGK Probed by NMR." Journal of Molecular Biology **330**(5): 1189-1201.

Sambrook, S., E. F. Fritsch and T. Maniatis (1989). Molecular Cloning: A Laboratory Manual. Cold Spring Harbor Laboratory Press, Cold Spring Harbor, NY.
